# A proof-of-concept study of pitolisant for excessive daytime sleepiness in patients with Prader-Willi syndrome

**DOI:** 10.5664/jcsm.11800

**Published:** 2025-11-01

**Authors:** Amee Revana, Rakesh Bhattacharjee, Jennifer L. Miller, Aaron Chidekel, Priya Khanna, Sarayu Ratnam, Grant Runyan, Eric Bauer, Krystle Davis Rapchak, David Seiden, Kumar Budur, Jeffrey M. Dayno

**Affiliations:** ^1^ Baylor College of Medicine, Texas Children’s Hospital, Houston, Texas; ^2^ Rady Children’s Hospital, University of California San Diego, San Diego, California; ^3^ University of Florida Health Shands Hospital, Gainesville, Florida; ^4^ Nemours Children’s Health, Wilmington, Delaware; ^5^ Ann & Robert H. Lurie Children’s Hospital of Chicago, Chicago, Illinois; ^6^ Harmony Biosciences, Plymouth Meeting, Pennsylvania

**Keywords:** behavioral symptoms, excessive daytime sleepiness, histamine H_3_ receptor, H_3_ histamine receptor antagonists, proof-of-concept study

## Abstract

**Study Objectives:**

The majority of patients with Prader-Willi syndrome experience excessive daytime sleepiness (EDS). This study evaluated the effects of pitolisant, a histamine 3 (H_3_)-receptor antagonist/inverse agonist that promotes wakefulness, in patients with Prader-Willi syndrome and EDS.

**Methods:**

In this phase 2, randomized, double-blind, placebo-controlled, proof-of-concept study, patients ages 6–65 years with a confirmed diagnosis of Prader-Willi syndrome with EDS were randomized 1:1:1 to receive lower-dose pitolisant (children/adolescents/adults, 8.9/13.35/17.8 mg), higher-dose pitolisant (children/adolescents/adults, 17.8/26.7/35.6 mg), or matching placebo for 11 weeks (3-week titration/8-week maintenance). The primary endpoint was change from baseline to week 11 in Epworth Sleepiness Scale for Children and Adolescents (parent/caregiver version) score. Other measures included the Caregiver Global Impression of Severity for EDS, Aberrant Behavior Checklist-Community, second edition, and Hyperphagia Questionnaire for Clinical Trials.

**Results:**

Of 65 patients randomized and treated, 59 (90.8%) completed the double-blind phase. Least-squares (LS) mean improvement from baseline to week 11 in Epworth Sleepiness Scale for Children and Adolescents score was greater for higher-dose pitolisant (−5.0) vs placebo (−3.9; LS mean [standard error] difference, −1.1 [1.52]), but not for lower-dose pitolisant (−3.5) vs placebo (LS mean [standard error] difference, 0.5 [1.6]). The largest effect of pitolisant was seen in children (ages 6 to < 12 years; LS mean [standard error] difference for higher-dose pitolisant vs placebo, −3.5 [1.90]). Improvements were observed across other measures, especially in the higher-dose pitolisant group, including LS mean (standard error) change of −5.5 (1.2) on the irritability domain of the Aberrant Behavior Checklist-Community, second edition, and −3.1 (1.0) on the Hyperphagia Questionnaire for Clinical Trials. The most common adverse events in pitolisant-treated patients (doses pooled) were anxiety, irritability, and headache (11.9% each), consistent with the known safety profile of pitolisant.

**Conclusions:**

Results of this proof-of-concept study support further evaluation of pitolisant in patients with Prader-Willi syndrome and EDS.

**Clinical Trial Registration:** Registry: ClinicalTrials.gov; Name: A Phase 2 Study to Evaluate the Safety and Efficacy of Pitolisant in Patients With Prader-Willi Syndrome, Followed by an Open Label Extension; URL: https://clinicaltrials.gov/study/NCT04257929; Identifier: NCT04257929.

**Citation:**

Revana A, Bhattacharjee R, Miller JL, et al. A proof-of-concept study of pitolisant for excessive daytime sleepiness in patients with Prader-Willi syndrome. *J Clin Sleep Med*. 2025;21(11):1893–1902.

**BRIEF SUMMARY**

**Current Knowledge/Study Rationale:** Excessive daytime sleepiness (EDS) is common in people with Prader-Willi syndrome, a genetic neurodevelopmental disorder characterized by hypothalamic dysfunction. Pitolisant is indicated for the treatment of EDS in patients with narcolepsy who are ≥ 6 years old, and reductions in EDS and behavioral problems have been observed in case studies of children and adolescents with Prader-Willi syndrome.

**Study Impact:** In this phase 2, exploratory, proof-of-concept study, treatment with pitolisant was associated with reductions in EDS and behavioral symptoms in patients ages 6–65 years with Prader-Willi syndrome and was well tolerated. These findings support further investigation of the efficacy and safety of pitolisant for patients with Prader-Willi syndrome and EDS in a larger placebo-controlled clinical trial.

## INTRODUCTION

Prader-Willi syndrome (PWS) is a rare genetic neurodevelopmental disorder characterized by hypothalamic dysfunction associated with a broad spectrum of features including hyperphagia, early onset childhood obesity, sleep abnormalities, behavioral problems, and cognitive impairment.^1^^,^^2^ The prevalence of PWS is approximately 1/10,000–1/20,000 individuals (range, 1/8,000–1/30,000), with an estimated 400,000 people with PWS worldwide, including 20,000 in the United States.^3^ Excessive daytime sleepiness (EDS) is a common, lifelong condition in people with PWS but is often underrecognized.^4^^–^^7^ In an analysis of data from the Global PWS Registry, EDS was reported in 378/690 patients (55%), with daytime sleepiness characterized as moderate or severe in 61% of patients at the time of reporting and 96% of patients at peak severity.^7^ EDS in patients with PWS often begins in early childhood and may continue across the lifespan.^4^^,^^5^^,^^7^ As a result, EDS can interfere with learning, adversely affect daily functioning and performance, and contribute to emotional disturbance and behavioral disorders.^5^^,^^8^ Increased napping and reduced daytime activity associated with EDS may also disrupt daily routines and adversely affect the quality of life of patients and their families.^5^

Many of the clinical manifestations of PWS are related to abnormalities in the development and function of the hypothalamus, which is a crucial region of the brain for regulating endocrine function, the balance between hunger and satiety, body temperature, and sleep-wake timing and stability.^2^^,^^9^ Further, key genes implicated in PWS (eg, *SNORD116* and *MAGEL2*) are abundantly expressed in the hypothalamus and are potential regulators of sleep in addition to food intake and energy balance.^2^^,^^10^^–^^13^

Histaminergic neurons are localized in the tuberomammillary nucleus of the hypothalamus and project to regions throughout the brain.^14^^,^^15^ The neurotransmitter histamine is involved in a wide range of physiological functions, including regulation of the sleep-wake cycle, food intake, arousal, cognition, and memory.^15^^–^^17^ Pitolisant (WAKIX; Harmony Biosciences, LLC; Plymouth Meeting, PA), a histamine 3 (H_3_)-receptor antagonist/inverse agonist, is approved by the United States Food and Drug Administration for the treatment of EDS and cataplexy in adults with narcolepsy and for the treatment of EDS in pediatric patients ≥ 6 years of age with narcolepsy.^18^ It is postulated that the wake-promoting effects of pitolisant are associated with increased synthesis and release of histamine via binding to presynaptic H_3_ autoreceptors and increased activity of other wake-promoting neurotransmitters by binding to H_3_ heteroreceptors on nonhistaminergic neurons.^17^^,^^19^ Previous case studies in children and adolescents with PWS treated with pitolisant reported reductions in EDS and behavioral problems and increased alertness, cognitive function, and engagement.^20^^–^^22^ This current study reports the results of a randomized, placebo-controlled, proof-of-concept study designed to evaluate the effects of pitolisant in the treatment of EDS in patients with PWS.

## METHODS

### Study design

**Figure 1:**
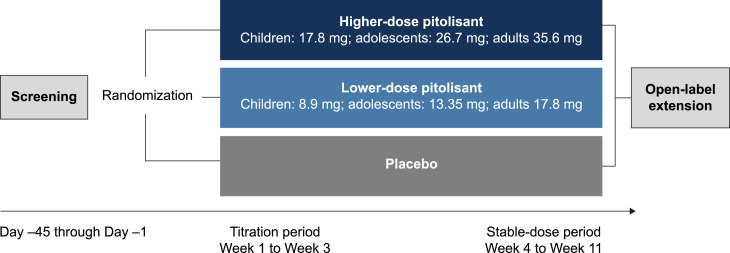
Study design.

This phase 2, randomized, double-blind, placebo-controlled, proof-of-concept study (ClinicalTrials.gov identifier, NCT04257929) was performed at 13 study centers across the United States. The study consists of a screening period (≤ 45 days), an 11-week double-blind treatment phase (including a 3-week titration period and an 8-week stable dose period), and an optional open-label extension (**Figure 1**). Results of the open-label extension will be reported separately. Study visits occurred during screening (day −45 to −2); at baseline (day −1); during the titration period at day 1 and weeks 1 and 2; and during the stable-dose period at weeks 3, 4, 5, 7, 8, and 11/end of treatment.

The study was approved by the independent ethics committee or institutional review board for each study center and was conducted in accordance with the principles of the Declaration of Helsinki, the International Council of Harmonisation guideline for Good Clinical Practice, and all applicable regulatory requirements. Patients and/or their legal guardians provided written informed consent/assent prior to the initiation of any study procedures.

### Patients

The study enrolled children (ages 6 to < 12 years), adolescents (ages 12 to < 18 years), and adults (ages 18–65 years) with a diagnosis of PWS confirmed by genetic testing and/or patient medical records. Other key inclusion criteria were adequate sleep duration (mean of ≥ 8 hours/night for children, ≥ 7 hours/night for adolescents, and ≥ 6 hours/night for adults), as demonstrated via patient sleep diary during screening, and EDS, as confirmed by a score ≥ 12 on the parent/caregiver version of the Epworth Sleepiness Scale for Children and Adolescents (ESS-CHAD) at the screening assessment. Exclusion criteria included a diagnosis of another genetic or chromosomal disorder distinct from PWS; history or presence of untreated obstructive sleep apnea (OSA) or high risk for sleep apnea, based on medical history and clinical assessment; another relevant underlying sleep disorder that was, in the opinion of the investigator, the primary contributing factor to EDS; the use of any investigational drug within 28 days before enrollment; and a history of substance abuse, a serious cardiovascular disorder, hepatic or renal abnormalities, or a psychiatric disorder.

### Treatment

Eligible patients were randomized in a 1:1:1 ratio using interactive response technology to receive lower-dose pitolisant (8.9 mg for children, 13.35 mg for adolescents, and 17.8 mg for adults), higher-dose pitolisant (17.8 mg for children, 26.7 mg for adolescents, and 35.6 mg for adults), or matching placebo. The doses for each age group were selected based on safety and efficacy data from the clinical development program for adults with narcolepsy and pharmacokinetic data from 24 pediatric patients with narcolepsy (ages 7 to < 18 years) who received a single dose of pitolisant. The pitolisant dose for adult patients is the same as that approved for the narcolepsy indication. The pediatric doses were chosen based on modeling using the available pediatric pharmacokinetic data, which suggest that pediatric patients have higher exposure to pitolisant than adults.

Patients were titrated to their randomized stable dose of study drug during the 3-week titration period and subsequently continued taking their randomized stable dose for an additional 8 weeks (**Table S1** in the supplemental material). Pitolisant tablets were provided in strengths of 4.45 mg and 17.8 mg, with matching placebo tablets for each tablet strength. Patients were instructed to take study drug once daily in the morning upon wakening, starting on the first day after the baseline visit (day 1). Patients/caregivers were instructed to record in the study diary the number of tablets administered from each bottle each day, and to record the time of study drug dosing on the day of visits at week 3 and week 11 and on the day before visit at week 7. Adherence to study drug administration was monitored by reviewing study diaries in conjunction with tablet counts of returned study medication. All patients/caregivers/parents/legal guardians, investigators, study site personnel, and sponsor staff were unaware of treatment assignments for the double-blind treatment phase.

### Assessments

The primary measure was the ESS-CHAD, a validated assessment tool for sleep propensity,^23^^,^^24^ as completed by a parent or caregiver. Additional secondary and exploratory assessments were the Caregiver Global Impression of Severity (CaGI-S) for EDS, the Aberrant Behavior Checklist-Community, second edition (ABC-C), and the Hyperphagia Questionnaire for Clinical Trials (HQ-CT), which was completed in conjunction with the Food Safe Zone Questionnaire. These assessments were completed at baseline and weeks 3, 7, and 11/end of treatment; lower scores were indicative of lower severity.

Safety was assessed by monitoring the incidence of adverse events (AEs) throughout the study and evaluating results of laboratory tests, vital signs, physical examinations, and 12-lead electrocardiograms (performed at screening, baseline, and weeks 3, 7, and 11/end of treatment). Each pediatric electrocardiogram was interpreted by a pediatric cardiologist. Suicidality was assessed using the Very Young Child/Cognitively Impaired–Lifetime Recent Columbia-Suicide Severity Rating Scale at screening and the Very Young Child/Cognitively Impaired–Since Last Contact Columbia-Suicide Severity Rating Scale at all other study visits. Anxiety was assessed using the Anxiety, Depression, and Mood Scale at screening, baseline, and weeks 3, 7, and 11/end of treatment.

### Statistical analysis

The planned sample size of 60 patients (lower-dose pitolisant group, n = 20; higher-dose pitolisant group, n = 20; placebo group, n = 20) was powered at 80% to detect a difference of 3.9 between the pooled pitolisant groups and the placebo group. Prior research suggested that the ESS-CHAD (parent/caregiver version) standard deviation falls between 5 and 6; thus, the proposed sample size would yield a 95% confidence interval of ± 2.7, with a standard deviation of 5, and ± 3.2, with an standard deviation of 6. This study was not powered to show statistical significance between treatment arms but, rather, was intended to determine whether there was an adequate efficacy signal to warrant further evaluation in a larger study. No formal adjustment for multiplicity was performed.

Measures of EDS, hyperphagia, and behavior were analyzed using the modified intent-to-treat (mITT) population, which included all patients who received at least 1 dose of study drug and had a baseline assessment and at least 1 postbaseline assessment for a given endpoint analysis during the double-blind treatment phase. Select endpoints were analyzed using the sensitivity efficacy population (SEP), which included all patients in the mITT population with the exception of 1 adolescent patient in the placebo group, who was considered an outlier due to having a baseline ESS-CHAD total score of 24 (the scale maximum) and a week 11 total score of 4, and having similarly unanticipated and extreme values on most other measures. Subgroup analyses based on age were performed for select endpoints.

The primary endpoint was the change from baseline to week 11 in ESS-CHAD score, as completed by a parent or caregiver, for pitolisant vs placebo, and the primary comparison of interest was the difference in the higher-dose pitolisant group vs placebo at week 11. A separate analysis evaluated treatment response rates, with response defined as a reduction of at least 3 points in the ESS-CHAD score or a final score of 10 or lower. Secondary endpoints included change from baseline to week 11 in CaGI-S and ABC-C scores. Change from baseline to week 11 in HQ-CT score was an exploratory endpoint. Findings for each endpoint were summarized using descriptive statistics. Least-squares (LS) means, standard errors (SEs), and the LS mean differences (each active treatment group vs placebo) are reported. Changes from baseline were analyzed using a mixed model for repeated measures (MMRM), with fixed effects included for treatment, visit, treatment-by-visit interaction, and baseline value. An unstructured covariance matrix was utilized and, if it failed to converge, first-order autoregressive and compound symmetry were attempted, in that order; Kenward-Roger degrees of freedom were used. While no explicit imputation was performed for the primary analysis, the MMRM was expected to yield unbiased results, under the assumption that data were missing at random.

Safety endpoints were analyzed in the safety population, which included all patients who received at least 1 dose of double-blind study treatment and were summarized using descriptive statistics. AEs were coded using the Medical Dictionary for Regulatory Activities version 25.0 and summarized by age group.

All analyses were performed using SAS 9.4 (SAS Institute, Cary, NC).

## RESULTS

### Patients

**Figure 2:**
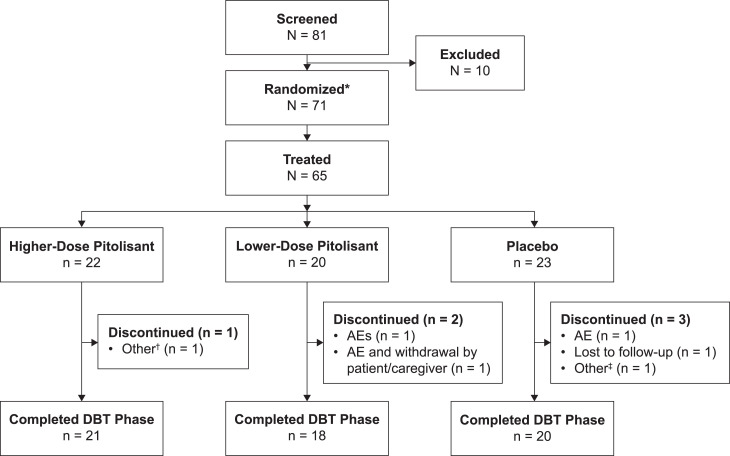
Patient disposition (double-blind treatment phase). *Six patients were randomized but did not receive the assigned treatment because they did not meet inclusion/exclusion criteria; †patient was diagnosed with/positive for COVID-19; ‡QTcF interval value of a single baseline ECG was > 442 ms (448 ms; mean of the triplicate was 428.3 ms and did not meet the exclusion criterion). AE = adverse event, DBT = double-blind treatment, ECG = electrocardiogram, QTcF = QT interval corrected for heart rate based on Fridericia’s formula.

Of 65 patients who were randomized and treated, 59 (90.8%) completed the double-blind treatment phase (**Figure 2**). All treated patients were included in the mITT and safety populations. Overall, the percentage of male (50.8%) and female (49.2%) patients was roughly equivalent, most patients were white (84.6%), and the mean (standard deviation) age was 12.4 (5.5) years.

Approximately half of the patients (52.3%) were in the youngest age group (6 to < 12 years). Notably, the maximum age across all patients was 28 years. Nineteen patients (29.2%) had comorbid OSA, for which they were receiving treatment as required for study enrollment. The patients with OSA allowed into the trial needed approval from their physicians to indicate that, based on medical history and clinical assessment, OSA was not the primary contributor to the patients’ EDS. Slight imbalances occurred across treatment groups for sex, race, age, and age group (**Table 1**). The median age of PWS diagnosis was within the first year of life for all treatment groups, but the mean age was lower in the placebo group relative to the pitolisant groups. The age at PWS diagnosis and type of PWS (data not shown) were consistent with the general patient population with PWS. In the overall population, the mean baseline ESS-CHAD score was 15.2 in the pooled pitolisant groups and 14.7 in the placebo group, reflecting EDS severe enough to warrant medical intervention (ESS-CHAD score > 10 is suggestive of EDS).^25^^–^^27^ The median rate of medication adherence was 97.4% in the pooled pitolisant groups and 95.1% in the placebo group.

**Table 1:** Demographic and baseline characteristics (mITT and safety populations).

	Higher-Dose Pitolisant (n = 22)	Lower-Dose Pitolisant (n = 20)	Placebo (n = 23)
Age, years			
Mean (SD)	13.1 (6.70)	12.1 (5.4)	11.9 (4.3)
Median (range)	11.0 (6–28)	11.0 (6–24)	12.0 (6–23)
Age group, n (%)			
6 to < 12 years	11 (50.0)	12 (60.0)	11 (47.8)
12 to < 18 years	6 (27.3)	4 (20.0)	9 (39.1)
18–65 years	5 (22.7)	4 (20.0)	3 (13.0)
Age at PWS diagnosis, years			
Mean (SD)	4.6 (7.8)	3.4 (6.5)	0.7 (2.9)
Median (range)	0.0 (0–26)	0.0 (0–22)	0.0 (0–14)
Female, n (%)	12 (54.5)	11 (55.0)	9 (39.1)
Race, n (%)			
White	21 (95.5)	17 (85.0)	17 (73.9)
Black or African American	0	1 (5.0)	0
Asian	1 (4.5)	1 (5.0)	4 (17.4)
Other	0	1 (5.0)	2 (8.7)
Hispanic or Latino ethnicity, n (%)	2 (9.1)	0	2 (8.7)
BMI, kg/m^2^, mean (SD)	26.7 (13.9)	25.5 (8.6)	24.2 (6.6)
Treated with somatropin, n (%)	18 (81.8)	17 (85.0)	22 (95.7)
Concomitant sleep disorders, n (%)	13 (59.1)	15 (75.0)	13 (56.5)
Baseline score, mean (SD)			
ESS-CHAD	14.8 (2.9)	15.7 (3.7)	14.7 (3.4)
CaGI-S for EDS	2.4 (0.8)	2.9 (0.9)	2.5 (1.0)
HQ-CT	8.4 (5.4)	15.1 (7.2)	12.3 (6.4)
ABC-C domain			
Irritability	11.6 (7.6)	15.4 (9.6)	13.8 (10.4)
Social withdrawal	7.9 (6.0)	8.1 (6.5)	7.0 (5.0)
Hyperactivity/nonadherence	10.6 (9.4)	9.8 (8.0)	10.7 (7.3)
Inappropriate speech	4.8 (3.4)	4.6 (3.2)	4.6 (3.2)
Stereotypic behavior	3.8 (3.5)	2.2 (3.2)	3.3 (4.5)

ESS-CHAD scores range from 0–24, with higher scores indicating an increase in likelihood of excessive daytime sleepiness. CaGI-S for EDS rates patient’s likelihood of falling asleep during daytime activities over the past week, on a scale from 0 (not at all) to 4 (very high likelihood). HQ-CT scores range from 0–36, with higher scores indicating increased hyperphagia. ABC-C domain scores range from 0–45 for irritability, 0 to 48 for social withdrawal and hyperactivity/nonadherence, 0–12 for inappropriate speech, and 0–21 for stereotypic behavior. Higher ABC-C domain scores indicate greater severity. ABC-C = Aberrant Behavior Checklist-Community, second edition, BMI = body mass index, CaGI-S = Caregiver Global Impression of Severity, EDS = excessive daytime sleepiness, ESS-CHAD = Epworth Sleepiness Scale for Children and Adolescents (parent/caregiver version), HQ-CT = Hyperphagia Questionnaire for Clinical Trials, mITT = modified intent-to-treat, PWS = Prader-Willi syndrome, SD = standard deviation.

### EDS

**Figure 3:**
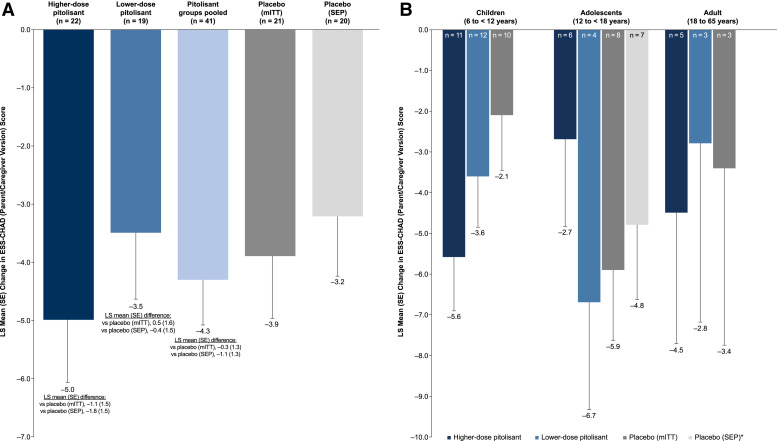
ESS-CHAD (parent/caregiver version). LS mean change from baseline to week 11 **(A)** in the overall population and **(B)** by age group (mITT population, unless otherwise indicated). *Based on a separate MMRM analysis with outlier excluded. ESS-CHAD scores range from 0–24, with higher scores indicating increased likelihood of excessive daytime sleepiness. ESS-CHAD = Epworth Sleepiness Scale for Children and Adolescents, LS = least-squares, mITT = modified intent-to-treat, MMRM = mixed model for repeated measures, SE = standard error, SEP = sensitivity efficacy population.

Mean ESS-CHAD parent/caregiver version total scores at baseline were similar for all treatment groups (**Table 1**). In the overall mITT population, LS mean change from baseline to week 11 in ESS-CHAD score was greater in patients treated with higher-dose pitolisant as well as in all patients pooled from lower-dose and higher-dose pitolisant groups as compared with patients in the placebo group (**Figure 3A**). The greatest change relative to placebo was in children, which was the cohort with the largest number of patients (**Figure 3B**). In children, the LS mean (SE) difference between the higher-dose pitolisant group and the placebo group was −3.5 (1.9). In the sensitivity analysis that excluded the outlier (see Methods), mean change scores on the ESS-CHAD were more consistent across age groups in the placebo group (**Figure 3B**). Among study completers, the response rate (the percentage of patients with improvement of ≥ 3 points from baseline or a week 11 score of ≤ 10) was higher for pitolisant (63.2%) vs placebo (52.6% overall and 50.0% with the outlier excluded), driven primarily by the higher-dose pitolisant group (70.0%) relative to the lower-dose group (55.6%).

In the overall mITT population, LS mean changes in CaGI-S scores from baseline to week 11 were similar across lower-dose pitolisant, higher-dose pitolisant, and placebo groups (**Figure S1** in the supplemental material). Reductions in CaGI-S scores were larger for pitolisant as compared with placebo in children (higher-dose pitolisant only) and adults (both doses). Exclusion of the outlier did not substantially affect the results in the overall and adolescent populations.

### Aberrant Behavior Checklist

Mean scores at baseline for each ABC-C domain were similar across lower-dose pitolisant, higher-dose pitolisant, and placebo groups (**Table 1**). In the overall population, LS mean ABC-C domain scores decreased from baseline to week 11 in both pitolisant treatment groups, with larger decreases observed with higher-dose pitolisant (**Table 2**). In particular, the largest LS mean (SE) decrease in the higher-dose pitolisant treatment group was observed for the irritability domain (−5.5 [1.16]). For both the mITT population and SEP, LS mean changes from baseline to week 11 in the higher-dose pitolisant treatment group were larger as compared to the placebo treatment group for all domains, with the exception of stereotypic behavior (**Table 2**). In particular, a greater LS mean reduction from baseline to week 11 on the inappropriate speech domain was observed for the higher-dose pitolisant group compared with the placebo group, both in the mITT population (LS mean [SE] difference, −1.7 [0.60]; *P =* .01) and the SEP (LS mean [SE] difference, −2.0 [0.58]; *P* < .01).

**Table 2:** Aberrant behavior checklist: LS mean (SE) change from baseline at week 11 (mITT population unless otherwise indicated).

Domain	Overall			Children (6 to < 12 Years)			Adolescents (12 to < 18 Years)			Adults (≥ 18 Years)		
	Higher-Dose Pitolisant (n = 22)	Lower-Dose Pitolisant (n = 20)	Placebo (mITT, n = 23)/ (SEP, n = 22)*	Higher-Dose Pitolisant (n = 11)	Lower-Dose Pitolisant (n = 11)	Placebo (n = 8)	Higher-Dose Pitolisant (n = 6)	Lower-Dose Pitolisant (n = 4)	Placebo (mITT, n = 8)/ (SEP, n = 7)*	Higher-Dose Pitolisant (n = 5)	Lower-Dose Pitolisant (n = 3)	Placebo (n = 3)
Irritability	−5.5 (1.2)	−2.1 (1.3)	−4.3 (1.2)/−2.9 (1.1)	−5.9 (1.4)	−2.0 (1.4)	−2.7 (1.6)	−4.9 (2.8)	−7.0 (3.4)	−5.9 (2.4)/−3.3 (21)	−3.2 (2.3)	−3.0 (2.7)	−3.1 (3.0)
Social withdrawal	−3.9 (0.7)	−1.4 (0.8)	−2.8 (0.7)/−2.4 (0.7)	−4.8 (0.8)	−1.3 (0.8)	−3.4 (0.9)	−3.1 (1.6)	−2.7 (2.0)	−3.0 (1.3)/−1.9 (1.3)	−3.4 (1.6)	−1.9 (1.8)	0.1 (2.2)
Hyperactivity/ nonadherence	−4.4 (1.0)	−0.2 (1.1)	−2.9 (1.1)/−1.8 (1.0)	−4.2 (1.1)	−0.6 (1.1)	−3.6 (1.2)	−4.2 (3.1)	−0.8 (3.8)	−3.0 (2.5)/−0.6 (1.8)	−5.0 (1.6)	1.7 (1.9)	−1.3 (2.1)
Inappropriate speech	**−1.9 (0.4)†‡**	−0.2 (0.4)	−0.2 (0.4)/0.0 (0.4)	−2.0 (0.4)	−0.1 (0.4)	−0.7 (0.5)	−1.4 (0.9)	−1.3 (1.1)	−0.8 (0.8)/−0.2 (0.7)	−2.2 (1.1)§	0.1 (1.4)	2.8 (1.4)
Stereotypic behavior	−1.0 (0.4)	−0.3 (0.4)	−1.2 (0.4)/−1.0 (0.4)	−0.9 (0.5)	−0.3 (0.5)	−0.8 (0.5)	−0.6 (1.2)	0.5 (1.5)	−1.1 (1.0)/−0.4 (0.9)	−1.5 (0.6)	−1.8 (0.8)	−2.2 (0.7)

Data shown are LS mean (SE) change. ABC-C domain scores range from 0–45 for irritability, 0–48 for social withdrawal and hyperactivity/nonadherence, 0–12 for inappropriate speech, and 0–21 for stereotypic behavior. Higher scores indicate greater severity. *Based on a separate MMRM analysis with outlier excluded; †*P* < .01 vs placebo (mITT); ‡*P* < .01 vs placebo (SEP); §*P* < .05 vs placebo (mITT). ABC-C = Aberrant Behavior Checklist-Community, second edition, LS = least-squares, mITT = modified intent-to-treat, MMRM = mixed model for repeated measures, SE = standard error, SEP = sensitivity efficacy population.

In the age-based subgroup analysis of ABC-C scores, greater score reductions in all domains except stereotypic behavior were observed in children in the higher-dose pitolisant group relative to the placebo group (**Table 2**). In adolescents, LS mean reductions from baseline to week 11 in the higher-dose pitolisant group were generally similar to those in the placebo group in the mITT population but greater than those in the placebo group after exclusion of the outlier (SEP). With the exception of the stereotypic behavior domain, LS mean reductions from baseline to week 11 in adults were larger with higher-dose pitolisant as compared to placebo, most notably in the inappropriate speech domain (LS mean [SE] difference, −5.0 [1.8]; *P* < .05).

### Hyperphagia

Mean HQ-CT score at baseline was 11.8 (out of a maximum [most severe] possible score of 36), indicating that most members of this study population did not have substantial hyperphagia.^28^ At baseline, the mean HQ-CT score in the higher-dose pitolisant group (8.4) was lower than the mean scores in the lower-dose pitolisant group (15.1) and the placebo group (12.3; **Table 1**). The LS mean decrease from baseline to week 11 in HQ-CT scores in the higher-dose pitolisant group was larger than that in the placebo treatment group for the SEP (**Figure S2** in the supplemental material). The LS mean decrease from baseline to week 11 was greater for both pitolisant groups vs placebo in children and for higher-dose pitolisant vs placebo in adults. In contrast, LS mean decreases in HQ-CT scores in adolescents were greater in the placebo group (including or excluding the outlier in the placebo group) compared to the pitolisant groups.

### Safety

The overall frequency of AEs was similar in the lower-dose pitolisant (65.0%) and placebo groups (65.2%) but was slightly lower in the higher-dose pitolisant group (50.0%) relative to placebo (**Table 3**). The most commonly reported AEs in patients receiving pitolisant were anxiety, irritability, and headache (11.9% each) compared with 8.7%, 4.3%, and 8.7%, respectively, in the placebo group. The overall incidence of AEs among pitolisant-treated patients was slightly lower in children (52.2%) than in adolescents (60.0%) and adults (66.7%). All AEs were mild or moderate in severity. Treatment-related AEs were reported in 26.2% of pitolisant-treated patients (higher dose, 18.2%; lower dose, 35.0%) and 30.4% of those receiving placebo (**Table 3**). The most common treatment-related AEs in the pooled pitolisant treatment group were anxiety (11.9%), irritability (9.5%), and headache (7.1%).

**Table 3:** Summary of adverse events (safety population).

AE, n (%)	Higher-Dose Pitolisant (n = 22)	Lower-Dose Pitolisant (n = 20)	Placebo (n = 23)
Any AE	11 (50.0)	13 (65.0)	15 (65.2)
Treatment-related AE*	4 (18.2)	7 (35.0)	7 (30.4)
Severe AE	0	0	0
Serious treatment-related AE	0	0	1 (4.3)
AE leading to discontinuation from study drug	1 (4.5)	2 (10.0)	2 (8.7)
AE leading to interruption of study drug	0	0	1 (4.3)
Most common AEs (≥ 2 patients in any treatment group)			
Anxiety	1 (4.5)	4 (20.0)	2 (8.7)
Irritability	1 (4.5)	4 (20.0)	1 (4.3)
Headache	2 (9.1)	3 (15.0)	2 (8.7)
COVID-19	3 (13.6)	0	2 (8.7)
Hyperphagia	1 (4.5)	2 (10.0)	2 (8.7)
Nasopharyngitis	2 (9.1)	2 (10.0)	1 (4.3)
Sleep disorder	0	2 (10.0)	0
Upper abdominal pain	0	2 (10.0)	0

* Considered by the investigator as possibly, probably, or definitely related to study drug. AE = adverse event.

No serious AEs were reported in pitolisant-treated patients. One patient in the placebo group experienced a serious AE of deep vein thrombosis that was considered unrelated to study drug. Five patients had AEs leading to discontinuation of study drug: 2 in the lower-dose pitolisant group (fine motor skill dysfunction, possibly related; and headache, possibly related), 1 in the higher-dose pitolisant group (hyperphagia, unlikely related), and 2 in the placebo group (COVID-19, not related; and anxiety, probably related). One patient in the placebo group had an AE (agitation) leading to interruption of study drug. No deaths were reported during the study.

Baseline values were similar across all treatment groups for each Anxiety, Depression, and Mood Scale domain total score, and decreases of similar magnitude from baseline to week 11 in mean domain total scores were observed across all treatment groups (**Table S2** in the supplemental material). No consistent, clinically meaningful trends in vital signs or laboratory parameters, suicidality, or worsening hyperphagia were reported. An AE of QT prolongation (moderate) considered treatment-related was reported for 1 patient in the placebo group. No clinically significant abnormal electrocardiogram findings were reported postbaseline in any pitolisant-treated patients.

## DISCUSSION

A growing body of evidence suggests that EDS is common in patients with PWS^4^^–^^7^ and can impact cognition, behavior, quality of life, and mental health in these individuals.^5^^,^^8^ While some patients with PWS also have sleep-disordered breathing, EDS in patients with PWS likely reflects the underlying pathophysiology of the disease because it does not consistently occur with (and can persist despite) treatment of sleep-disordered breathing.^5^^,^^29^ Currently, no medications are approved specifically to treat EDS in patients with PWS, and management relies on off-label use of stimulants and other wake-promoting treatments, such as pitolisant and modafinil.^11^

This phase 2, exploratory, proof-of-concept study is the first randomized clinical trial to evaluate the effects of pitolisant as a treatment for EDS in PWS. At baseline, all patients had EDS that was substantial enough to warrant intervention (score ≥ 12 on the ESS-CHAD). A greater decrease in ESS-CHAD (parent/caregiver version) total scores was observed in the higher-dose pitolisant treatment group compared with placebo. The difference between groups was larger after excluding the outlier (see Methods), with the resulting value (−1.8) approaching the clinical significance threshold (based on an American Academy of Sleep Medicine meta-analysis^30^) of ≥ 2 points. Overall, 70.0% of patients receiving higher-dose pitolisant were classified as responders on the ESS-CHAD (based on an improvement of ≥ 3 points from baseline or a score ≤ 10).

Reductions in hyperphagia (as measured by the HQ-CT) were seen in patients who received higher-dose pitolisant, even though baseline scores were nominally low. Histamine has been previously described as playing a role in the regulation of food intake.^16^ Specifically, activation of postsynaptic H_1_ receptors has been shown to decrease food intake, whereas H_1_ antagonists (ie, antihistamines) are associated with increased food intake and weight gain.^16^ Because H_3_ receptor antagonists/inverse agonists, such as pitolisant, bind to H_3_ autoreceptors on histamine neurons, which increases synaptic levels of histamine, they have the potential to suppress food consumption.^16^ Thus, reductions in hyperphagia may be due to direct effects of pitolisant on food intake, as well as indirect effects mediated by reductions in daytime sleepiness.

In this study, reductions in negative behaviors assessed by the ABC-C (irritability, social withdrawal, hyperactivity/nonadherence, inappropriate speech) were larger with higher-dose pitolisant compared to placebo. Previous research found an association between daytime sleepiness and emotional/behavioral problems in children, adolescents, and young adults with PWS, such that greater daytime sleepiness corresponded with worse emotional/behavioral disturbance.^31^ This association was independent of sleep-disordered breathing or adequacy of nighttime sleep duration.^31^ The reductions in irritability observed in the present study are notable, as irritability is a common clinical feature of PWS that can be exacerbated by daytime sleepiness.^8^^,^^32^ As relationships between sleep and behavioral problems (including stereotypic behaviors and communication issues) have also been observed in autism spectrum disorder,^33^^,^^34^ and histamine has been identified as a contributor to tic disorders (including Tourette’s syndrome),^35^ behavioral improvements in pitolisant-treated patients with PWS may be associated with changes in histamine neurotransmission and/or reductions in daytime sleepiness; however, further investigation is required.

Although this proof-of-concept study was not powered to detect statistically significant differences between pitolisant and placebo, reductions in daytime sleepiness, hyperphagia, and behavioral symptoms were observed in pitolisant-treated patients. Such changes in people with PWS may provide long-term health benefits (eg, increased participation in daily activities, decreased food intake, more opportunities for socialization), which warrant evaluation in future studies. To the extent that EDS promotes sedentary behaviors, reductions in daytime sleepiness may help decrease body weight.

Subgroup analyses based on age showed that the activity for higher-dose pitolisant vs placebo across multiple measures was pronounced in the 6 to < 12 years age group. A clinically meaningful difference (−3.5) from baseline to week 11 in ESS-CHAD (parent/caregiver version) was seen, as well as greater activity on CaGI-S scores measuring EDS severity, HQ-CT scores measuring hyperphagia, and the irritability domain of the ABC-C. These changes are noteworthy, given the limited sample size overall and the lack of enrichment criteria for the ABC-C or HQ-CT assessments required for eligibility. The pronounced activity in children for some measures may be related to dosing, as this age group received the highest milligram per kilogram dose of pitolisant on average. In addition, children generally showed a smaller placebo response as compared with the adolescent and adult age groups and comprised the largest number of patients, which may have facilitated detection of drug-placebo differences. Reductions in daytime sleepiness, hyperphagia, and behavioral symptoms may be particularly beneficial for children with PWS, as earlier intervention provides a greater opportunity to improve outcomes.

The overall safety profile of pitolisant in patients with PWS in this study was consistent with the established safety profile of pitolisant in patients with narcolepsy, with no new safety signals observed.^18^ Subgroup analyses, based upon age groups, showed that the safety profile of pitolisant was similar in children, adolescents, and adults with PWS. No evidence of QT prolongation was observed among patients receiving pitolisant in the current study.

This study was limited by small sample size, due to its exploratory design, and was not powered to detect statistically significant differences between treatment arms. A parallel-group design was chosen because a 2-period crossover design would require a considerably longer duration (ie, 11 weeks for each period, plus additional time for a washout phase between the 2 periods), increasing the burden on patients and caregivers suffering from a very challenging disease and increasing the risk of dropout. Due to difficulties with continuous positive airway pressure adherence in the PWS population,^8^^,^^36^ formal adherence testing was not undertaken Additionally, imbalances across treatment groups with regard to sex, race, age, age group, and baseline HQ-CT scores limit interpretation of the results.

## CONCLUSIONS

EDS is common in people with PWS and can negatively affect functioning. In patients with PWS ages 6–65 years, pitolisant reduced EDS. A dose-response relationship was observed, with the higher-dose pitolisant group showing greater reductions from baseline in ESS-CHAD scores at the end of the study. Behavioral symptoms (as measured by the ABC-C) also decreased, especially in the higher-dose pitolisant group. Some reductions in hyperphagia were noted, even though baseline hyperphagia scores were in the normal/mild range. Treatment with pitolisant was well tolerated in patients with PWS, with no new safety findings. Based on the positive signals from this phase 2 study, a phase 3 clinical trial (TEMPO; NCT06366464) is ongoing, to further evaluate the safety and efficacy of pitolisant in the treatment of EDS and other symptoms in patients ≥ 6 years of age with a confirmed diagnosis of PWS.

## DISCLOSURE STATEMENT

All authors have seen and approved the manuscript. A.R. reports serving as an investigator for Harmony Biosciences and a consultant to TREND, LLC. R.B. reports serving as a consultant to Avadel Pharmaceuticals, Harmony Biosciences, Jazz Pharmaceuticals, and the Institute for Advanced Clinical Trials; and receiving speaker fees for Harmony Biosciences. J.L.M. reports receiving research funding from Harmony Biosciences, Rhythm Pharmaceuticals, Soleno Therapeutics, and TRYP Therapeutics. A.C. reports serving as a consultant to BioMarin Pharmaceuticals. P.K. and S.R. report nothing to disclose. G.R., E.B., K.D.R., D.S., K.B., and J.D. are employees of Harmony Biosciences, Plymouth Meeting, PA. Editorial and medical writing assistance was provided under the direction of the authors by Adrienne Drinkwater, PhD, and Pratibha Hebbar, PhD, Synchrony Medical Communications, LLC, West Chester, PA, and funded by Harmony Biosciences, LLC, Plymouth Meeting, PA.

## OPEN ACCESS

Copyright 2025 The Authors. This is an open access article, distributed under the Creative Commons Attribution 4.0 International License. Sharing and adaptation are permitted provided attribution to its original publication in the Journal of Clinical Sleep Medicine is made in accordance with the license.

## Supplemental Materials

10.5664/jcsm.11800 Supplemental Materials

## ABBREVIATIONS

ABC-C: Aberrant Behavior Checklist-Community, second edition
AE: adverse event
CaGI-S: Caregiver Global Impression of Severity
EDS: excessive daytime sleepiness
ESS-CHAD: Epworth Sleepiness Scale for Children and Adolescents
H_3_: histamine 3
HQ-CT: Hyperphagia Questionnaire for Clinical Trials
LS: least-squares
mITT: modified intent-to-treat
PWS: Prader-Willi syndrome
SE: standard error
SEP: sensitivity efficacy population
