# Supplementary material for: A proof-of-concept study of pitolisant for excessive daytime sleepiness in patients with Prader-Willi syndrome
Source: J Clin Sleep Med. 2025 Nov 1;21(11):1893–902. doi: 10.5664/jcsm.11800 (PMC12582198; doi:10.5664/jcsm.11800)
Supplement: Supplementary file 1 [file jcsm.11800.sm001.pdf]

Table S1—Dosing in the double-blind treatment phase.

Age/Treatment Groups	Titration Period (3 weeks)			Stable Dose Period (8 weeks)
	**Week 1 (Days 1–7)**	**Week 2 (Days 8–14)**	**Week 3 (Days 15–21)**	**Weeks 4–11 (Days 22–77)**
**Children (6 to < 12 Years)**				
Lower-dose pitolisant	4.45 mg	8.9 mg	8.9 mg	8.9 mg
Higher-dose pitolisant	4.45 mg	8.9 mg	17.8 mg	17.8 mg
Placebo	Matching tablets	Matching tablets	Matching tablets	Matching tablets
**Adolescents (12 to < 18 Years)**				
Lower-dose pitolisant	4.45 mg	8.9 mg	13.35 mg	13.35 mg
Higher-dose pitolisant	8.9 mg	17.8 mg	26.7 mg	26.7 mg
Placebo	Matching tablets	Matching tablets	Matching tablets	Matching tablets
**Adults (18 to 65 Years)**				
Lower-dose pitolisant	4.45 mg	8.9 mg	17.8 mg	17.8 mg
Higher-dose pitolisant	8.9 mg	17.8 mg	35.6 mg	35.6 mg
Placebo	Matching tablets	Matching tablets	Matching tablets	Matching tablets

Table S2—Change from baseline to Week 11 in ADAMS domain total scores (safety population).

Domain	Pitolisant			Placebo
	Pooled	Higher-Dose	Lower-Dose	
**Manic/Hyperactive Behavior, Mean (SD), n**				
Baseline	5.6 (3.2), 42	5.8 (3.0), 22	5.5 (3.5), 20	5.6 (2.8), 23
CFB to Week 11	–1.4 (2.2), 38	–2.0 (1.8), 20	–0.9 (2.4), 18	–2.3 (2.7), 20
**Depressed Mood, Mean (SD), n**				
Baseline	8.1 (3.1), 42	7.9 (3.1), 22	8.4 (3.1), 20	8.3 (3.8), 23
CFB to Week 11	–2.3 (2.9), 38	–3.6 (2.6), 20	–0.8 (2.6), 18	–2.2 (4.3), 20
**Social Avoidance, Mean (SD), n**				
Baseline	5.0 (4.4), 42	4.5 (4.2), 22	5.5 (4.8), 20	5.2 (3.1), 23
CFB to Week 11	–1.4 (2.2), 38	–1.9 (2.2), 20	–1.0 (2.1), 18	–1.9 (3.7), 20
**General Anxiety, Mean (SD), n**				
Baseline	6.9 (4.4), 42	6.9 (4.6), 22	7.0 (4.3), 20	7.9 (4.2), 23
CFB to Week 11	–2.4 (2.7), 38	–2.9 (2.6), 20	–1.9 (2.9), 18	–2.5 (3.1), 20
**Obsessive/Compulsive Behavior, Mean (SD), n**				
Baseline	4.6 (2.8), 42	4.9 (2.6), 22	4.4 (30), 20	4.3 (2.3), 23
CFB to Week 11	–1.3 (2.3), 38	–1.4 (2.2), 20	–1.2 (2.5), 18	–0.6 (2.2), 20

ADAMS = Anxiety, Depression, and Mood Scale, CFB = change from baseline, SD = standard deviation.

Figure S1—CaGI-S for EDS: LS mean change from baseline at Week 11 (mITT population, unless otherwise indicated).

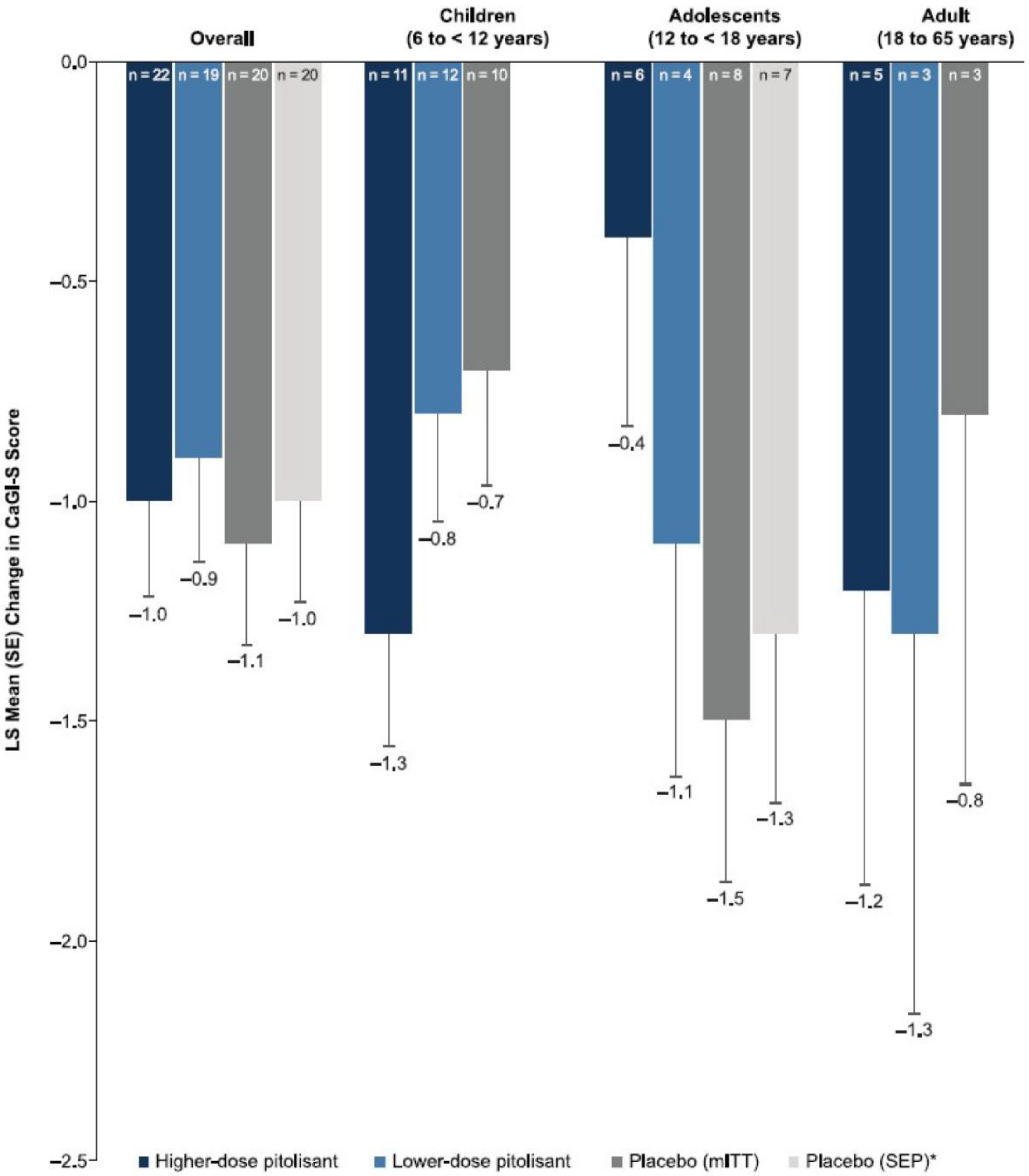

*Based on a separate MMRM analysis with outlier excluded. CaGI-S for EDS rates patient's likelihood of falling asleep during daytime activities over the past week, on a scale from 0 (not at all) to 4 (very high likelihood).

CaGI-S = Caregiver Global Impression of Severity, EDS = excessive daytime sleepiness, LS = least-squares, mITT = modified intent-to-treat, MMRM = mixed model for repeated measures, SE = standard error, SEP = sensitivity efficacy population.

Figure S2—HQ-CT: LS mean change from baseline at Week 11 (mITT population, unless otherwise indicated).

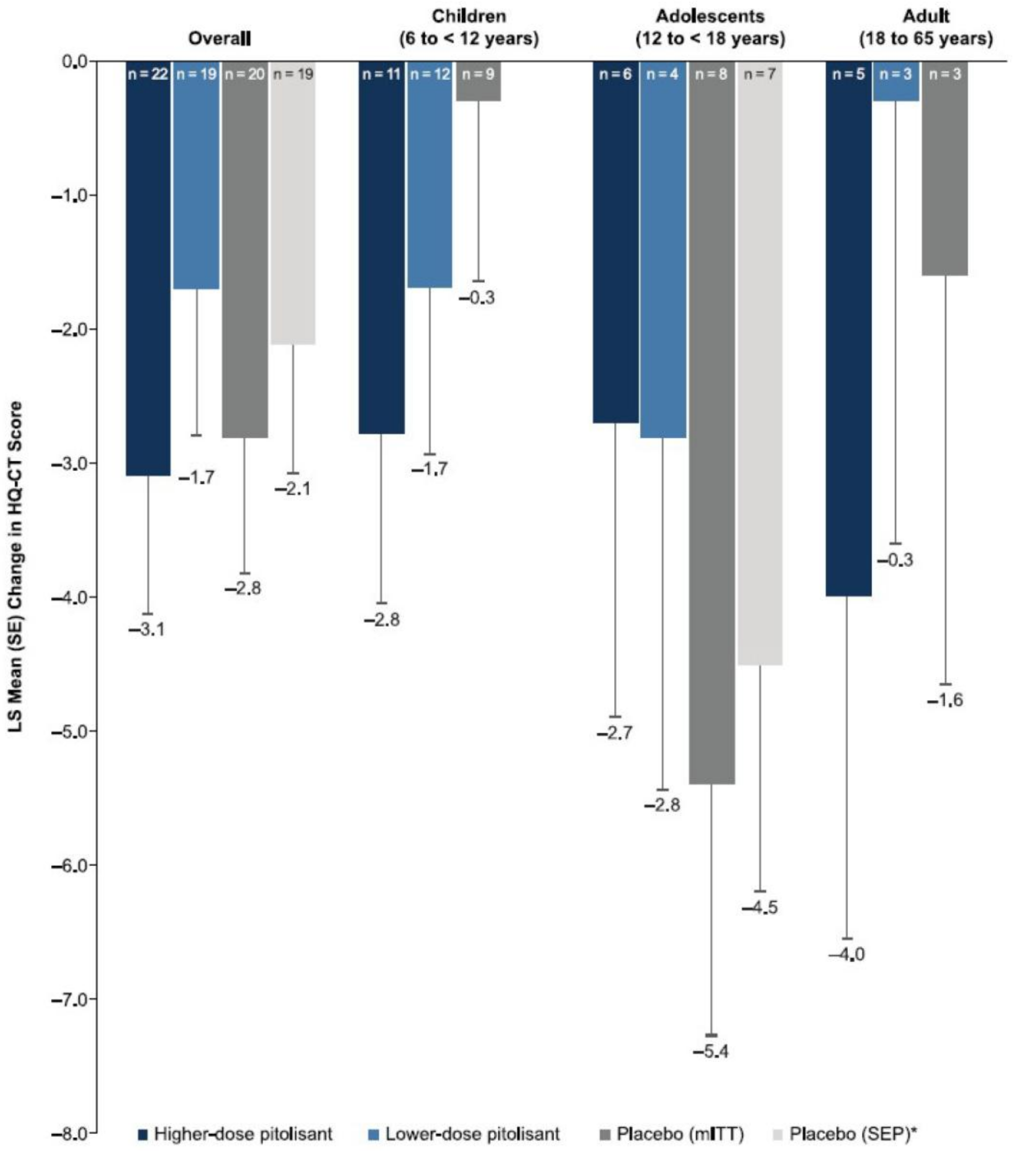

*Based on a separate MMRM analysis with outlier excluded. HQ-CT scores range from 0 to 36, with higher scores indicating increased hyperphagia.[1] Scores of approximately 11 to 13 are considered indicative of moderate hyperphagia that may be amenable to treatment.
HQ-CT = Hyperphagia Questionnaire for Clinical Trials, LS = least-squares, mITT = modified intent-to-treat, MMRM = mixed model for repeated measures, SEP = sensitivity efficacy population.

Reference

1. McCandless SE, Yanovski JA, Miller J, et al. Effects of MetAP2 inhibition on hyperphagia and body weight in Prader-Willi syndrome: a randomized, double-blind, placebo-controlled trial. *Diabetes Obes Metab*. 2017;19(12):1751-1761.
